# Supplementary material for: Alpibectir–Ethionamide combination (AlpE) for the treatment of tuberculosis
Source: Nat Commun. 2026 Apr 7;17:4954. doi: 10.1038/s41467-026-71460-6 (PMC13234193; doi:10.1038/s41467-026-71460-6)
Supplement: Supplementary file 1 — Supplementary Information [file 41467_2026_71460_MOESM1_ESM.pdf]

SUPPLEMENTARY INFORMATION

Alpibectir–Ethionamide (AlpE) combination for the treatment of tuberculosis

Zainab Edoo[1,2,#], Camille Grosse[1,3,#], Thomas Maitre[4,5,#], Rosangela Frita[1,2,#], Aurélie Chauffour[4], Laure Fournier Le Ray[4], Alexandre Godmer[4,6], Alexandra Aubry[4,7], Marilyne Bourotte[2], Rudy Antoine[1], Lina Tawk[1], Stéphanie Slupek[1], Vincent Trebosc[8], Birgit Schellhorn[8], Aurore Dreneau[2], Line Hofmann[2], Christian Kemmer[8], Sergio Lociuro[2,8], Glenn E. Dale[2,8], Françoise Jung[8], Esther Pérez-Herrán[9], Alfonso Mendoza[9], Maria Jose Rebollo López[9], Sonja Ghidelli-Disse[10], Thilo Werner[10], Lluis Ballell[9], David Barros Aguirre[9], Vanessa Mathys[11], Karine Soetaert[11], Véronique Megalizzi[3], René Wintjens[3], Marc Gitzinger[2,8], Benoit Deprez[12,13,§], Nicolas Veziris[4,6], Modesto J Remuiñán[9,§], Nicolas Willand[12,§,*], Michel Pieren[8,§,*], Alain R. Baulard[1,13,§,*]

[1] Univ. Lille, CNRS, Inserm, Institut Pasteur de Lille, U1019 - UMR 9017 - CIIL - Center for Infection and Immunity of Lille, Lille, France.

[2] BioVersys SAS, Lille, France.

[3] Research Department in Drug Development, Faculty of Pharmacy, Université Libre de Bruxelles, ULB, Bruxelles, Belgium

[4] Sorbonne Université, Inserm, Centre d'Immunologie et des Maladies Infectieuses, CIMI, F-75013 Paris, France.

[5] APHP. Sorbonne Université, Hôpital Tenon, Service de Pneumologie et d'Oncologie Thoracique, Centre de Référence Maladie Rares, GRC Sorbonne Université SOLID, Paris, France.

[6] APHP. Sorbonne Université, Hôpital Saint-Antoine, Département de Bactériologie, Centre National de Référence des Mycobactéries, Paris France

[7] APHP. Sorbonne-Université, Hôpital Pitié Salpêtrière, Laboratoire de Bactériologie-Hygiène, Centre National de Référence des Mycobactéries et de la résistance des mycobactéries aux antituberculeux, Paris France

[8] BioVersys AG, Basel, Switzerland.

[9] GSK, Tres Cantos R&D, PTM, Tres Cantos, 28760 Madrid, Spain.

[10] Cellzome GmbH. A GSK Company, 69117 Heidelberg, Germany

[11] National Reference Center for Tuberculosis and Mycobacteria, Sciensano, Brussels, Belgium.

[12] Univ. Lille, Inserm, Institut Pasteur de Lille, U1177 - Drugs and Molecules for Living Systems, Lille, France.

[13] Univ. Lille, CNRS, Inserm, CHU Lille, Institut Pasteur de Lille, US 41 - UMS 2014 - PLBS, F-59000 Lille, France

[#] These authors contributed equally: Zainab Edoo, Camille Grosse, Thomas Maitre, Rosangela Frita
[§] These authors jointly supervised this work: Benoit Deprez, Modesto J Remuiñán, Nicolas Willand, Michel Pieren, Alain Baulard
* email to: nicolas.willand@univ-lille.fr ; michel.pieren@bioversys.com ; alain.baulard@pasteur-lille.fr

Supplementary Table 1. Transcriptome and proteome profiles of *M. tuberculosis* H37Rv treated with alpibectir alone or combined with Eto.

Pathway	Gene name	Identifier	Protein description	Treatment with alpibectir		
				mRNA LFC		Protein LFC
				alone	+ Eto	
virS/mymA	*sadH*	*rv3085*	oxidoreductase	105.3	116.6	17.8
virS/mymA	*mymA*	*rv3083*	oxidoreductase	89.7	126.3	21.4
virS/mymA	*lipR*	*rv3084*	acetyl-hydrolase/esterase	87.9	128.3	9.4
virS/mymA	*adhD*	*rv3086*	zinc-type alcohol dehydrogenase	52.1	90.4	10.9
virS/mymA	*rv3087*	*rv3087*	diacylglycerol acyltransferase	47.6	95.4	14.5
virS/mymA	*tgs4*	*rv3088*	triacylglycerol synthase	40.0	88.9	6.6
virS/mymA	*fadD13*	*rv3089*	chain-fatty-acid-CoA ligase	34.6	77.5	13.7
virS/mymA	*virS*	*rv3082c*	virulence-regulating transcriptional regulator	7.6	15.7	23.5
	rv3599c	*rv3599c*	hypothetical protein	5.3	0.6	ND
	pqqE	*rv0693*	probable coenzyme PQQ synthesis protein E	5.0	1.8	1.6
	rv2862A	*rv2862A*	possible antitoxin	4.8	0	ND
	fucA	*rv0727c*	possible L-fuculose phosphate aldolase	4.4	1.0	1.0
	rv2306A	*rv2306A*	possible membrane protein	4.2	2.3	ND
	rv0692	*rv0692*	hypothetical protein	4.2	2.4	ND
ethA2/ethR2	*ethA2*	*rv0077c*	oxidoreductase	3.8	7.5	5.3
	rv0691A	*rv0691A*	mycofactocin precursor protein	3.7	2.0	ND
	rv1706A	*rv1706A*	hypothetical protein	3.3	0	ND
	rv2654c	*rv2654c*	possible PhiRv2 prophage protein	3.3	0.5	ND
	rv1927	*rv1927*	hypothetical protein	3.1	1.4	ND
ethA2/ethR2	*ethR2*	*rv0078*	transcriptional regulator	0.7	1.4	1.2
ethA/ethR	*ethA*	*rv3854c*	monooxygenase	1.1	0.9	0.1
ethA/ethR	*ethR*	*rv3855*	transcriptional repressor	1.1	0.7	0.4

Only genes with a linear fold-change (LFC) of at least 3 in the case of "alpibectir alone" are shown except for *ethR2*, *ethA*, and *ethR*, which are shown for information purposes even if their LFC is less than < 3. ND, not detected. For transcriptomics profile of alpibectir alone, n = 3. For transcriptomics profile of alpibectir + Eto, n = 1. For proteomics, n = 2.

Supplementary Table 2. Data collection and refinement statistics of alpibectir/VirS complex structure

Data Collection[a]	
PDB id	pdb_00008RCX
Synchrotron beamline	ESRF/MASSIF-1
Wavelength (Å)	0.9655
Space group	P61
Unit cell parameters (Å)	115.31, 115.31, 70.71
Resolution range (Å)	19.24-1.49 (1.53-1.49)
Completeness (%)	96.3 (92.4)
No. of reflections	487062 (25148)
No. of unique reflections	78828 (3979)
Multiplicity	6.2 (6.4)
R_{meas}[b]	0.172 (0.965)
$\langle I/\sigma(I)\rangle$[c]	6.5 (6.4)
$CC_{1/2}$[d]	0.997 (0.106)
Refinement statistics	
Number of reflections[e]	74849 (3501)
Number of refined atoms	
Protein	3173
Water	275
Other	44
Final R_{work}[f]/R_{free}[g] (%)	18.0/20.4
RMSD bond lengths (Å)	0.0149
RMSD bond angles (°)	2.129
Overall mean B factor ($Å^2$)	21.16
MolProbity statistics	
Ramachandran favoured (%)	96.80
Ramachandran outliers (%)	0.49
Clash score all-atom	2.98 (98th percentile)
MolProbity score	1.44 (88th percentile)
Alpibectir density fit analysis	
RSR	0.062
RSCC	0.977
RSZO	4.8
RSZD	1.05

[a] For data-collection, the numbers in parentheses represent values for the highest resolution shell. The data processing was performed with autoPRO[1] and STARANISO[2].

[b] $R_{meas} = \sum_{hkl} [N/(N-1)]^{½} \sum_i |I_i(hkl) - \langle I(hkl)\rangle| / \sum_{hkl} \sum_i I_i(hkl)$, is the multiplicity (N) independent R_{merge}, where I_i is the intensity of the ith observation and $\langle I\rangle$ is the mean intensity of the reflections.

[c] $\langle I/\sigma(I)\rangle$ = mean of $I/\sigma(I)$ of unique reflections.

[d] The mean intensity correlation coefficient of half-datasets.

[e] Numbers in parentheses are the number of reflections in the free set.

[f] $R_{work} = \sum||F_{obs}| - |F_{calc}||/\sum|F_{obs}|$, where F_{calc} and F_{obs} are the calculated and observed structure factor amplitude, respectively.

[g] $R_{free} = \sum||F_{obs}| - |F_{calc}||/\sum|F_{obs}|$, where all reflections belong to a test set of 5% randomly selected data.

Supplementary Table 3. Susceptibility of *M. tuberculosis* strains mutated in Eto activation pathways to Eto, Eto-SO, Pto, or Pto-SO in the absence or presence of 0.33 mg/L of alpibectir. Values were obtained from 2 technical replicates from 1 of 3 biological replicates. Source data are provided as a Source Data file.

Strain	Activation pathway affected	IC_{50} (mg/L)							
		Eto		Eto-SO		Pto		Pto-SO	
		alone	+ alpibectir	alone	+ alpibectir	alone	+ alpibectir	alone	+ alpibectir
H37Rv	none	1.18	0.03	3.52	0.04	0.31	0.03	0.44	0.03
8F	VirS/MymA	6.58	8.46	8.26	11.91	1.75	2.83	2.16	7.54
Tn3083	VirS/MymA	4.17	11.80	8.62	10.28	1.39	2.23	1.56	9.52
Tn3854	EthR/EthA	19.89	0.02	12.66	0.03	2.12	0.03	5.03	0.03
CD6R1	EthR/EthA and VirS/MymA	>50	>50	>50	>50	>50	>50	>50	>50

Supplementary Table 4. Frequency of spontaneous resistance of *M. tuberculosis* H37Rv to the alpibectir/Eto combination. Source data are provided as a Source Data file. n = 1.

Eto (mg/L)	Alpibectir (mg/L)	FoR
1	0.052	$1x10^{-5}$
1	0.107	$1x10^{-5}$
1	0.213	$1x10^{-5}$
0.5	0.107	$3x10^{-6}$
0.5	0.213	$1x10^{-5}$
0.375	0.052	$1x10^{-5}$

Supplementary Table 5. Mutations found in the spontaneous resistant mutants to the alpibectir/Eto combination. wt, wild-type gene; *, stop codon; fs, frameshift mutation.

	ethA	*mymA*	*virS*	Selection condition
Mutant-2	wt	Asp319fs	wt	Eto (0.5 mg/L) + alpibectir (0.107 mg/L)
Mutant-3	His22Asn	Tyr50*	wt	Eto (1 mg/L) + alpibectir (0.052 mg/L)
Mutant-4	wt	Asp319fs	wt	Eto (1 mg/L) + alpibectir (0.107 mg/L)
Mutant-5	Gly343Arg	wt	wt	Eto (1 mg/L) + alpibectir (0.107 mg/L)
Mutant-6	Ala247Pro	wt	wt	Eto (1 mg/L) + alpibectir (0.213 mg/L)
Mutant-7	Tyr392Ala	Tyr131*	wt	Eto (1 mg/L) + alpibectir (0.213 mg/L)
Mutant-8	Thr88Ile	wt	wt	Eto (0.375 mg/L) + alpibectir (0.052 mg/L)
Mutant-11	Gly343Arg	wt	wt	Eto (0.5 mg/L) + alpibectir (0.213 mg/L)
Mutant-12	Tyr408fs	wt	wt	Eto (1 mg/L) + alpibectir (0.052 mg/L)
Mutant-13	wt	Tyr145*	wt	Eto (1 mg/L) + alpibectir (0.107 mg/L)
Mutant-14	wt	Asn247fs	wt	Eto (1 mg/L) + alpibectir (0.107 mg/L)
Mutant-15	wt	wt	Tyr106*	Eto (1 mg/L) + alpibectir (0.213 mg/L)
Mutant-16	wt	wt	Tyr106*	Eto (1 mg/L) + alpibectir (0.213 mg/L)
Mutant-19	Arg279*	Tyr64*	wt	Eto (0.5 mg/L) + alpibectir (0.052 mg/L)
Mutant-20	Phe157Cys	Phe5fs	wt	Eto (0.5 mg/L) + alpibectir (0.107 mg/L)
Mutant-21	Gln360*	wt	wt	Eto (0.5 mg/L) + alpibectir (0.213 mg/L)
Mutant-22	Ser148Pro	wt	wt	Eto (1 mg/L) + alpibectir (0.052 mg/L)
Mutant-23	wt	wt	Tyr106*	Eto (1 mg/L) + alpibectir (0.107 mg/L)
Mutant-27	wt	Tyr50*	wt	Eto (0.5 mg/L) + alpibectir (0.052 mg/L)

Supplementary Table 6. BD BACTEC MGIT960 monitoring of the growth inhibition of *M. tuberculosis* H37Rv by alpibectir/Eto combinations. Bacterial growth inhibition is deduced from its remaining capacity to respirate in the presence of various concentrations of Eto or alpibectir/Eto.

Controls	Eto (mg/L)	Alpibectir (mg/L)			
		0	0.017	0.05	0.1
1% (GC)	0	G			
100% (GC)	0	G			
	0		G	G	G
	0.031		NG	NG	NG
	0.063		NG	NG	NG
	0.125		NG	NG	NG
	0.25		NG	NG	NG
	0.5	G	NG	NG	NG
	1	NG	NG	NG	NG
	2	NG			
	4	NG			

Supplementary Table 7A. Number of mice and lung CFU counts (mean of log_{10} ± SD) in each experimental group. Source data are provided as a Source Data file.

Groups	Log_{10} CFU			Number of mice	Number of mice dead before the date of sacrifice	Number of mice excluded from the analysis (culture contamination)
	D1	D14	D28			
D1	5.4 ± 0.3			12	0	1
Untreated*			8.9 ± 0.2	12	12	0
INH (25 mg/kg)		4.6 ± 0.5	4.4 ± 0.6	6 (D14)<br>6 (D28)	0 (D14)<br>0 (D28)	0
Eto (5 mg/kg)			7.8 ± 0.8	6	3	0
Eto (15 mg/kg)		6.3 ± 0.6	5.9 ± 0.7	6 (D14)<br>6 (D28)	0 (D14)<br>0 (D28)	0 (D14)<br>0 (D28)
Eto (50 mg/kg)		5.3 ± 0.7	4.7 ± 0.3	6 (D14)<br>6 (D28)	0 (D14)<br>0 (D28)	0 (D14)<br>0 (D28)
Eto (100 mg/kg)			4.9 ± 0.5	6	1**	2
Eto (200 mg/kg)			4.2 ± 0.3	6	0	0 (D14)<br>0 (D28)
Alpibectir (0.1 mg/kg)			8.2 ± 0.5	6	5	1
Alpibectir (0.5 mg/kg)			8.1 ± 0.3	6	5	2
Alpibectir (1.6 mg/kg)		6.6 ± 0.4	6.8 ± 0.3	6 (D14)<br>6 (D28)	0 (D14)<br>0 (D28)	1 (D14)<br>0 (D28)
Eto (5 mg/kg) + Alpibectir (0.1 mg/kg)			6.3 ± 0.5	7	0	1
Eto (15 mg/kg) + Alpibectir (0.1 mg/kg)		5.8 ± 0.2	5.0 ± 1.1	6 (D14)<br>6 (D28)	0 (D14)<br>0 (D28)	0 (D14)<br>1 (D28)
Eto (50 mg/kg) + Alpibectir (0.1 mg/kg)			4.8 ± 0.3	6	0	0
Eto (5 mg/kg) + Alpibectir (0.5 mg/kg)			5.0 ± 0.6	6	0	1
Eto (15 mg/kg) + Alpibectir (0.5 mg/kg)		4.8 ± 0.5	3.9 ± 0.8	6 (D14)<br>6 (D28)	1 (D14)<br>0 (D28)	2 (D14)<br>0 (D28)
Eto (50 mg/kg) + Alpibectir (0.5 mg/kg)			4.1 ± 1.1	6	1	1
Eto (5 mg/kg) + Alpibectir (1.6 mg/kg)			5.5 ± 0.6	6	0	0
Eto (15 mg/kg) + Alpibectir (1.6 mg/kg)		4.6 ± 0.5	3.7 ± 1.1	6 (D14)<br>6 (D28)	1 (D14)<br>0 (D28)	2 (D14)<br>0 (D28)
Eto (50 mg/kg) + Alpibectir (1.6 mg/kg)			4.2 ± 1.5	6	0	0

* Untreated mice died between D12 and D26.
** The culture medium was contaminated (i.e., excluded from CFU analysis).

Supplementary Table 7B. Lung CFU counts (mean in $\log_{10}$ ± SD) and DCFU in the experimental groups. Source data are provided as a Source Data file.

Treatment	D1	D14	D to D1	D28	D to D1	p-value[1]
Pre-treatment	5.4 ± 0.3				-	-
Untreated*				8.9 ± 0.2	3.5	
INH (25 mg/kg)		4.6 ± 0.5	-0.8	4.4 ± 0.6	-1.0	
Eto (5 mg/kg)				7.8 ± 0.9	2.4	
Eto (15 mg/kg)		6.3 ± 0.6	0.9	5.9 ± 0.7	0.5	
Eto (50 mg/kg)		5.3 ± 0.7	0.0	4.7 ± 0.4	-0.6	
Eto (100 mg/kg)				4.9 ± 0.5	-0.5	
Eto (200 mg/kg)				4.2 ± 0.3	-1.2	p = 0.02
Alpibectir (0.1 mg/kg)				8.2 ± 0.6	2.8	
Alpibectir (0.5 mg/kg)				8.1 ± 0.3	2.8	
Alpibectir (1.6 mg/kg)		6.6 ± 0.4	1.3	6.8 ± 0.3	1.5	
Eto (5 mg/kg) + Alpibectir (0.1 mg/kg)				6.3 ± 0.5	0.9	
Eto (15 mg/kg) + Alpibectir (0.1 mg/kg)		5.8 ± 0.2	0.5	5.0 ± 1.1	-0.4	
Eto (50 mg/kg) + Alpibectir (0.1 mg/kg)				4.8 ± 0.3	-0.6	
Eto (5 mg/kg) + Alpibectir (0.5 mg/kg)				5.0 ± 0.6	-0.3	
Eto (15 mg/kg) + Alpibectir (0.5 mg/kg)		4.8 ± 0.5	-0.6	3.9 ± 0.8	-1.4	p = 0.002
Eto (50 mg/kg) + Alpibectir (0.5 mg/kg)				4.1 ± 1.2	-1.2	p = 0.02
Eto (5 mg/kg) + Alpibectir (1.6 mg/kg)				5.5 ± 0.6	0.1	
Eto (15 mg/kg) + Alpibectir (1.6 mg/kg)		4.6 ± 0.5	-0.8	3.7 ± 1.1	-1.6	p = 0.0002
Eto (50 mg/kg) + Alpibectir (1.6 mg/kg)				4.2 ± 1.5	-1.2	p = 0.02

* Untreated mice died between D12 and D26.

[1]Anova (two-sided), Dunnett's posttest. Compared to pretreatment (D1), $p < 0.05$ was considered significant

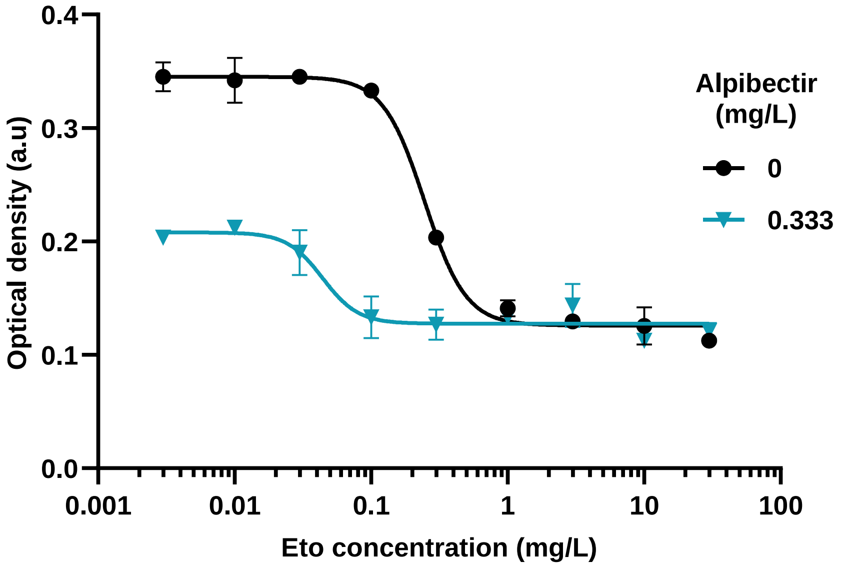

Supplementary Figure 1. OD measurement to determine susceptibility of H37Rv to Eto in combination with alpibectir. Growth reduction of H37Rv with Eto alone or in combination with 0.333 mg/L of alpibectir as measured by optical density. Data points represent the mean +/- standard deviation of 2 technical replicates. Results shown are representative of 3 biological replicates. Source data are provided as a Source Data file.

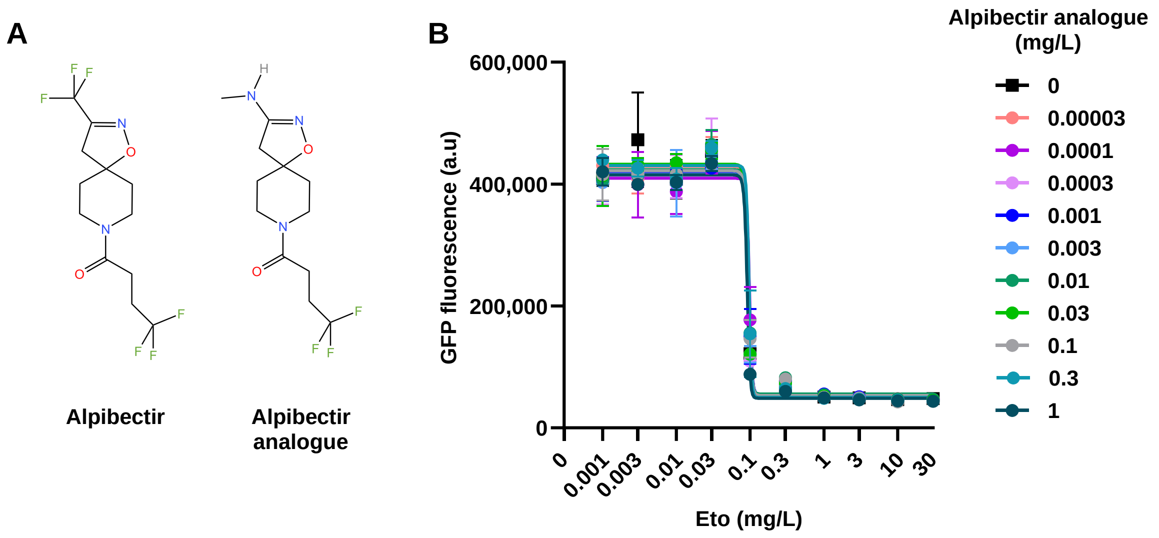

Supplementary Figure 2. (A) Structure of alpibectir and its analogue. Synthesis of the analogue will be described elsewhere (manuscript in preparation). **(B)** Concentration-response curves obtained for Eto alone or in the presence of increasing concentrations of the alpibectir analogue against H37Rv. Results shown are representative of 3 biological replicates. Data points represent the mean ± standard deviation of 2 technical replicates. Source data are provided as a Source Data file.

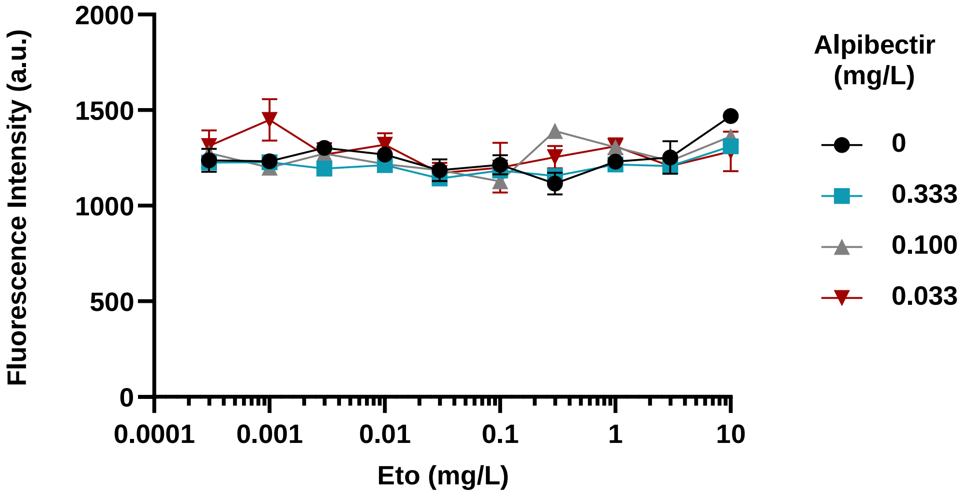

Supplementary Figure 3. THP-1 cells are viable for the duration of antibiotic treatment irrespective of drug concentrations. The fluorescence of THP-1 cells was measured after 5 days of incubation with the corresponding antibiotic concentrations. Results shown are representative of 3 biological replicates. Data points represent the mean +/- standard deviation of up to 2 technical replicates. Source data are provided as a Source Data file.

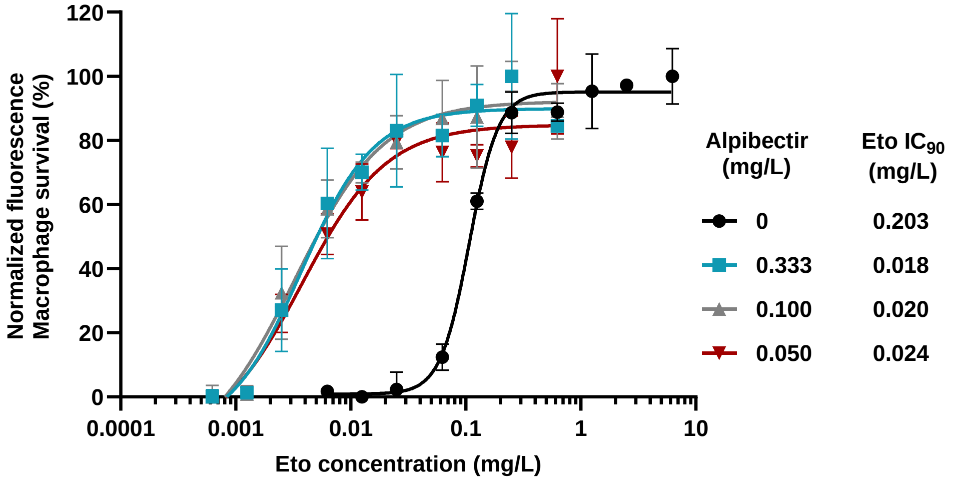

Supplementary Figure 4. Eto concentration-response curves and IC_{90} of Eto alone or in combination with alpibectir against intracellular H37Rv in a macrophage survival assay (multiplicity of infection = 10). Macrophage survival was measured using resazurin after 5 days of incubation with the corresponding drug treatment. In this assay, the efficacy of drugs to prevent infection-induced macrophage lysis is measured. The intrinsic activity of alpibectir did not prevent macrophage lysis. Results shown are representative of 3 biological replicates. Data points represent the mean +/- standard deviation of 2 technical replicates. Source data are provided as a Source Data file.

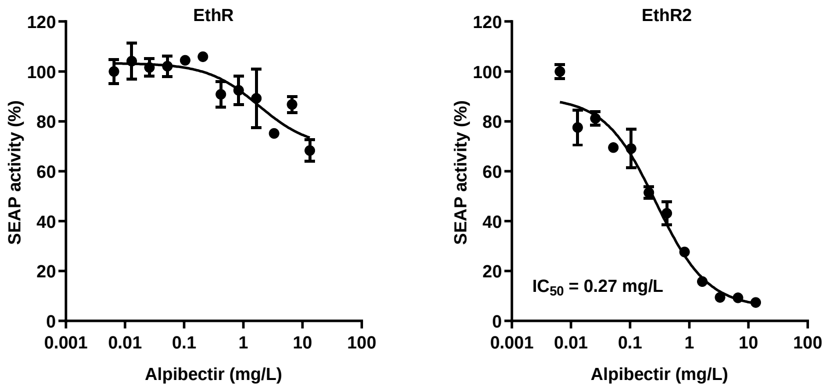

Supplementary Figure 5. Concentration-dependent inhibition of the binding of EthR or EthR2 to their cognate DNA operator region in the presence of varying concentrations of alpibectir. DNA binding was measured by the expression of the SEAP reporter gene. The IC_{50} values are indicated when applicable. Data points represent the mean +/- standard deviation of 2 technical replicates. Source data are provided as a Source Data file.

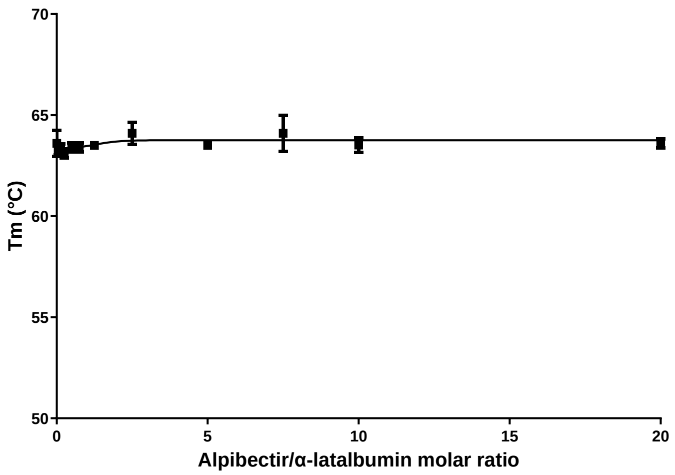

Supplementary Figure 6. The thermal denaturation of α-latalbumin was monitored using SYPRO Orange in the presence of increasing concentrations of alpibectir. The fluorescence signal was plotted as a function of temperature and the melting point (Tm) of α-latalbumin was obtained using the first-derivative of the fluorescence as a function of temperature (-dF/dT) and was plotted as a function of ligand/protein molar ratio. Data points represent the mean +/- standard deviation of 5 technical replicates. Source data are provided as a Source Data file.

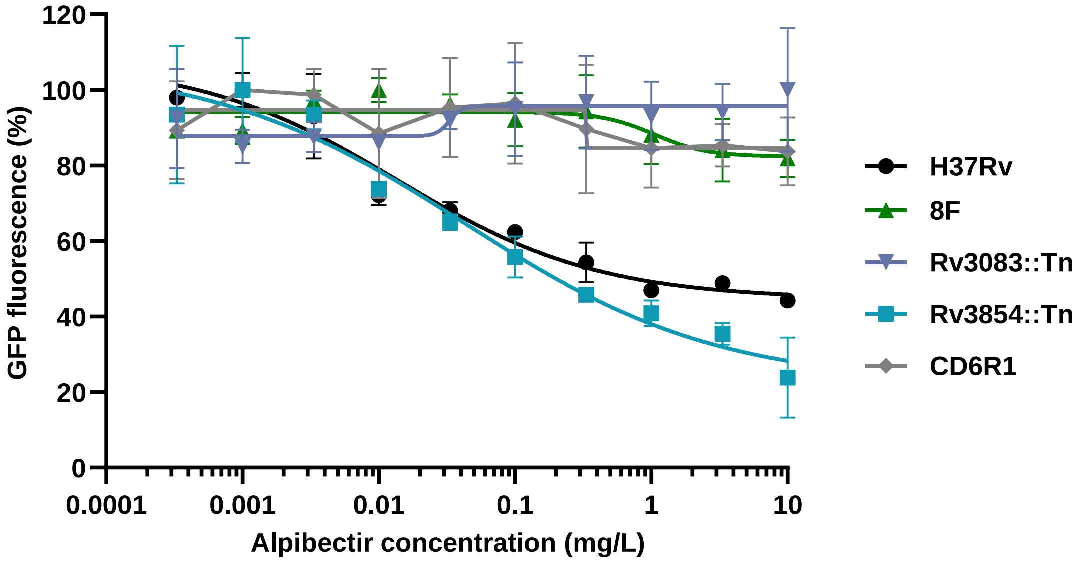

Supplementary Figure 7. Antibacterial activity of alpibectir alone on *M. tuberculosis* H37Rv and on strains mutated in Eto activation pathways. Percentage fluorescence was calculated using as the maximum mean fluorescence of each strain at the lowest alpibectir concentration. Data points represent the mean +/- standard deviation of 2 technical replicates. Results are representative of 3 biological replicates. Source data are provided as a Source Data file.

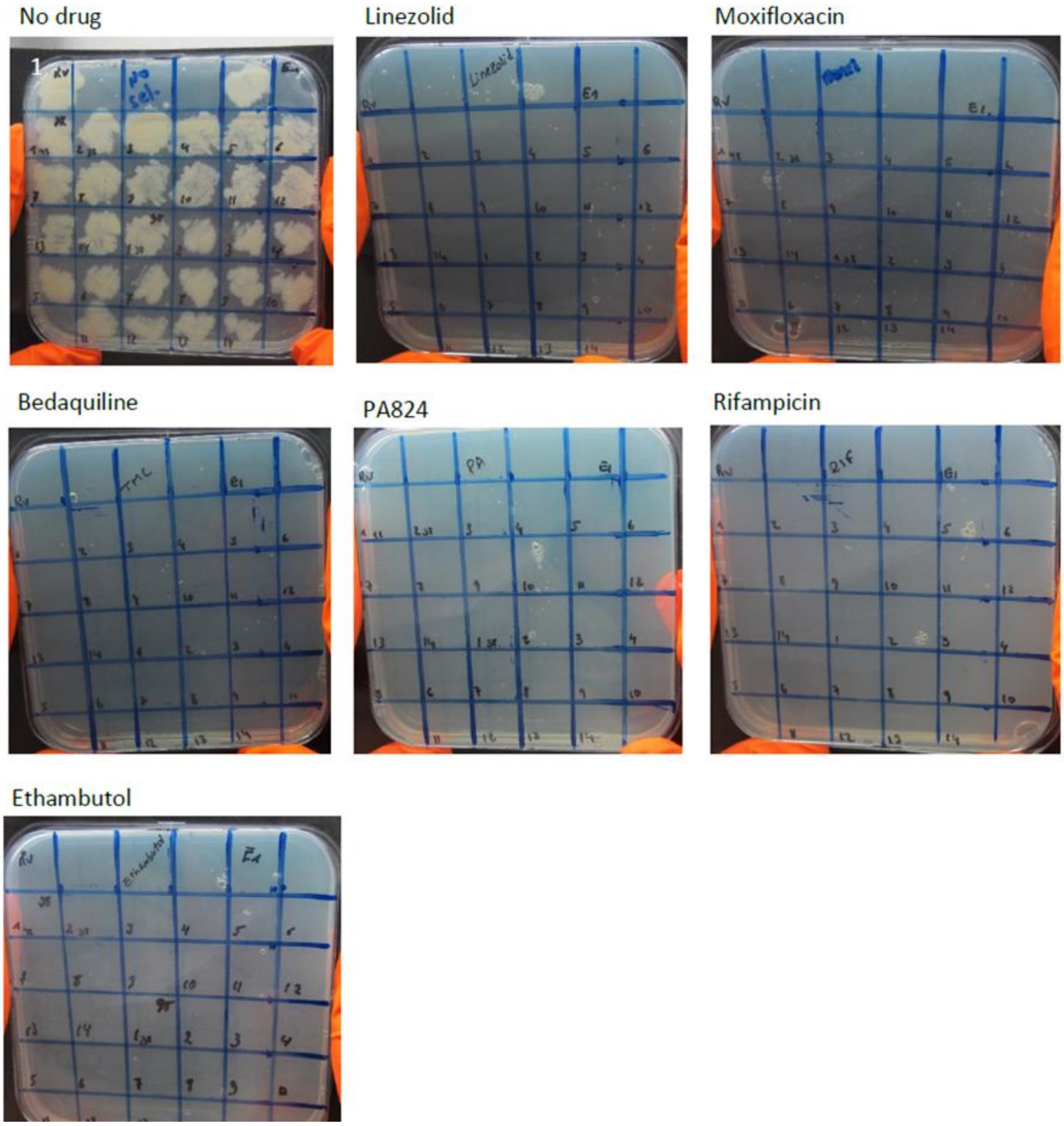

Supplementary Figure 8. Cross-resistance between the alpibectir/Eto combination and some standard-of-care anti-TB drugs. Fourteen clones that developed resistance to the alpibectir/Eto combination were tested for potential cross-resistance with moxifloxacin (1 mg/L), linezolid (1 mg/L), ethambutol (4 mg/L), bedaquiline (0.5 mg/L), pretomanid (PA824, 2 mg/L) and rifampicin (0.5 mg/L). These concentrations correspond to 2x the MIC or, whenever available, 2x the epidemiological cut-off (ECOFF; representing measures of a drug MIC distribution that separate bacterial populations into those representatives of a WT population and those with acquired or mutational resistance to the drug). Wild-type H37Rv and Eto-resistant E1 were used as controls.

**Ethionamide (Eto)**	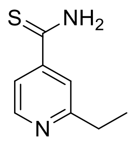
**Ethionamide sulfoxide (Eto-SO)**	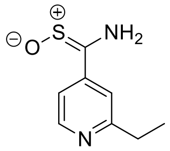
**Prothionamide (Pto)**	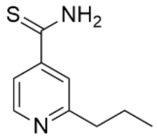
**Prothionamide sulfoxide (Pto-SO)**	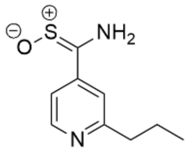

Supplementary Figure 9. The chemical structures of Eto, Eto-SO, Pto and Pto-SO.

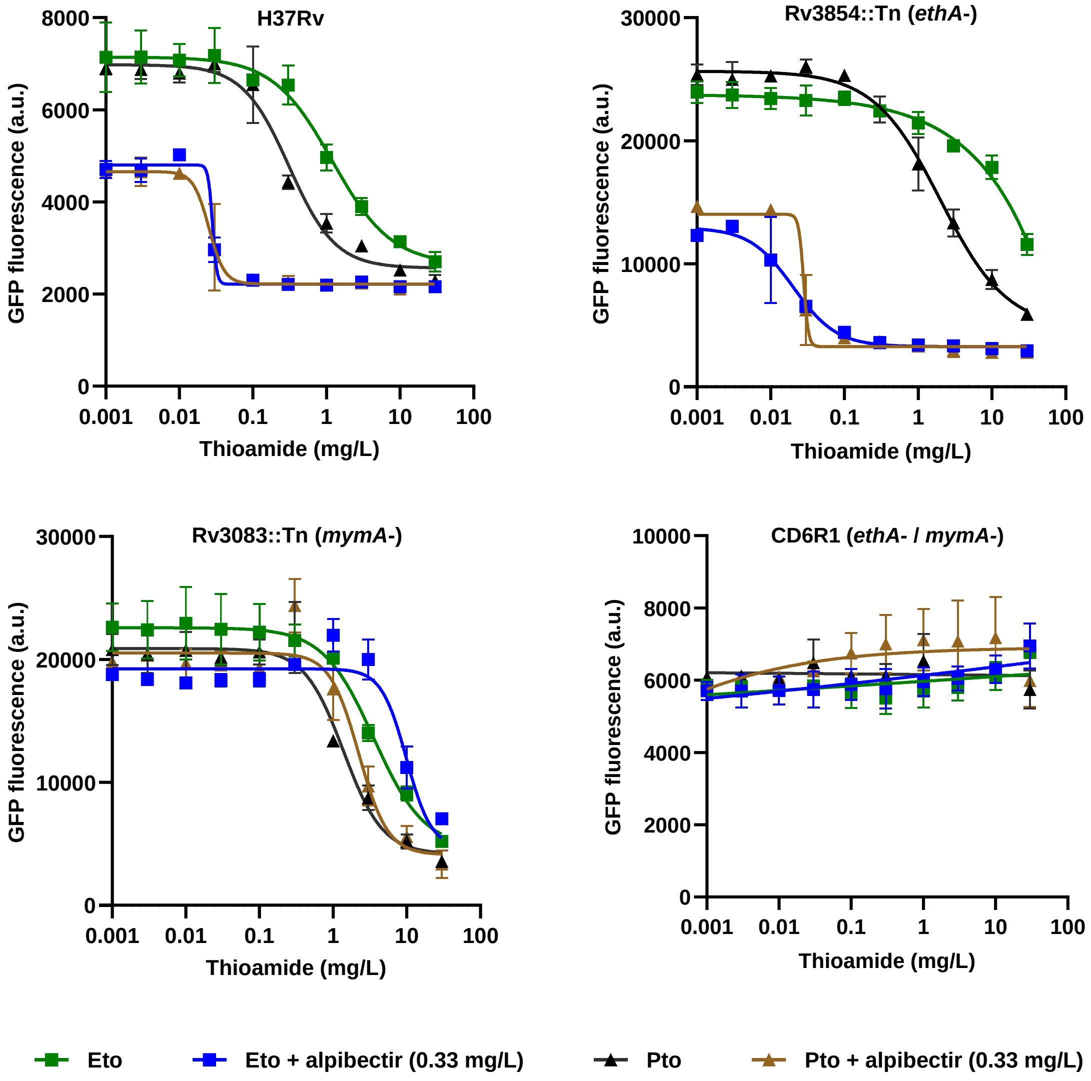

Supplementary Figure 10. Antibacterial activity of thioamides Eto or Pto alone or in the presence of alpibectir (0.33 mg/L) against *M. tuberculosis* H37Rv and strains mutated in the activation pathway(s) of Eto: 8F, Tn3083, Tn3854, and CD6R1. The mutated genes in each strain are indicated. Data points represent the mean +/- standard deviation of 2 technical replicates. Results shown are representative of 3 biological replicates. Source data are provided as a Source Data file.

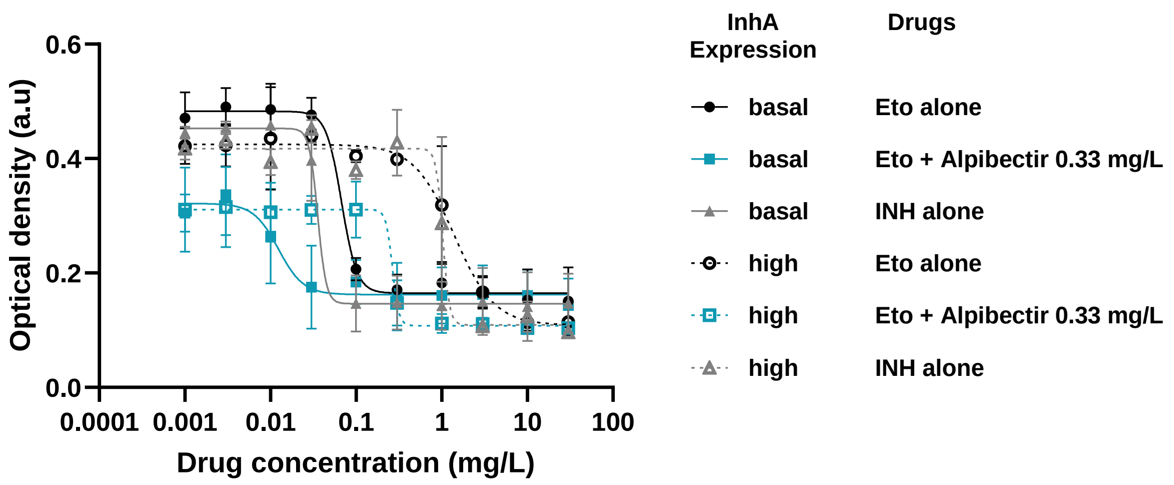

Supplementary Figure 11. Antibacterial activity of Eto alone, INH alone and of AlpE against *M. tuberculosis* expressing InhA at basal or high levels. Optical density was measured after 7 days of incubation with the corresponding drug concentrations. Data points show the normalized mean +/- standard deviation of 2 technical replicates from 1 of 3 biological replicates. Source data are provided as a Source Data file.

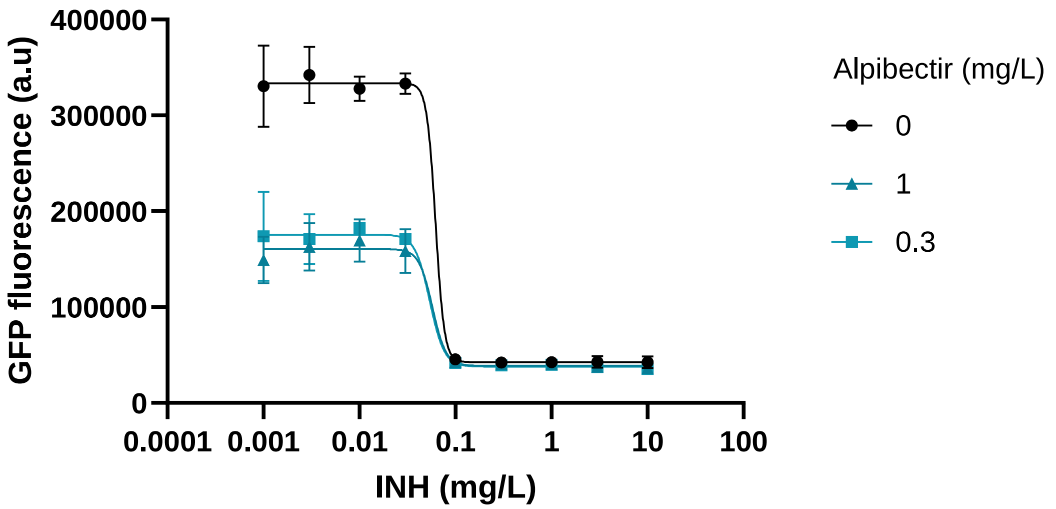

Supplementary Figure 12. Antibacterial activity of INH alone or in the presence of alpibectir against *M. tuberculosis* H37Rv. GFP fluorescence was measured after 5 days of incubation with the corresponding drug concentrations. Data points show the normalized mean +/- standard deviation of 2 technical replicates from 1 of 3 biological replicates. Source data are provided as a Source Data file.

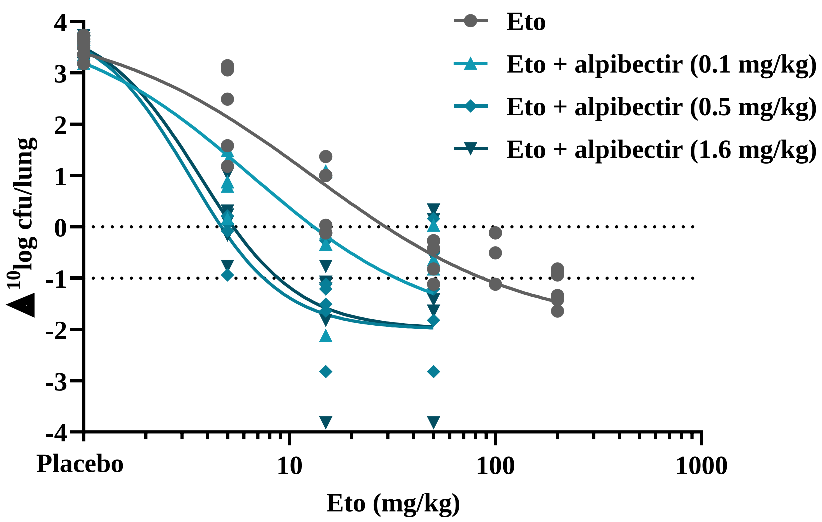

TDD (mg/kg)	Bacteriostasis	1-log_{10} killing
Eto	29.2	86.4
Eto + alpibectir (0.1 mg/kg)	13.1	33
Eto + alpibectir (0.5 mg/kg)	4.7	7.5
Eto + alpibectir (1.6 mg/kg)	5.3	8.8

Supplementary Figure 13. Dose-effect analysis of Eto with or without alpibectir in a 4-week treatment in BALB/c infected mice. Non-linear regression (variable slope, 4 parameters). TDD, total daily dose. Source data are provided as a Source Data file.

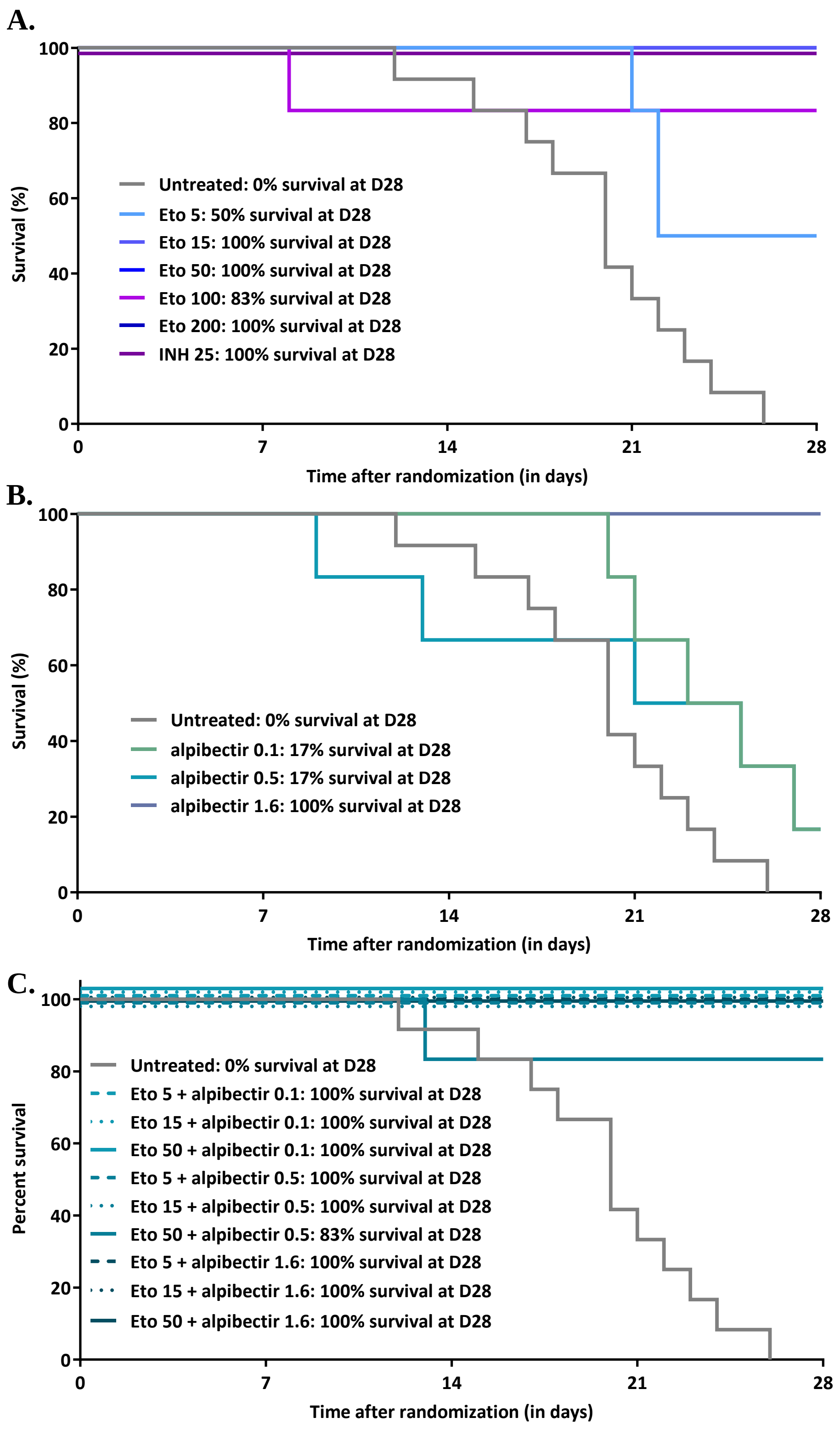

Supplementary Figure 14. Survival analysis at D28 in mice treated with (A) INH, and Eto alone, (B) alpibectir alone (preventive model) and (C) alpibectir/Eto. Source data are provided as a Source Data file.

References

1. Vonrhein C, Flensburg C, Keller P, Sharff A, Smart O. Paciorek W, Womack T & Bricogne G. Data processing and analysis with the autoPROC toolbox. *Acta Cryst*. **67**, 293-302 (2011).
2. Tickle IJ, Flensburg C, Keller P, Paciorek W, Sharff A, Vonrhein C & Bricogne G. STARANISO. Cambridge, United Kingdom: Global Phasing Ltd. (2018-2022).
