# Supplementary material for: Alpibectir–Ethionamide combination (AlpE) for the treatment of tuberculosis
Source: Nat Commun. 2026 Apr 7;17:4954. doi: 10.1038/s41467-026-71460-6 (PMC13234193; doi:10.1038/s41467-026-71460-6)
Supplement: Supplementary file 2 — Description of Additional Supplementary Files [file 41467_2026_71460_MOESM2_ESM.pdf]

Title: Supplementary Data 1

Description: MS/MS data for whole-proteome profiling of Mycobacterium tuberculosis treated with alpibectir, and vehicle control. Mycobacterium tuberculosis was treated for 72h with alpibectir 0.33mg/L or vehicule. Samples were processed and analyzed in parallel to generate whole proteome profiling data. The experiment was performed in duplicate for both the alpibectir-treated and vehicle control conditions. Foldchange ratios were calculated from sum ion areas of samples and compared to one vehicle control that was set to 1. SSM; number of spectrum-to-sequence matches, UPM; unique peptide matches, NA; not available, NBF; nonbound fraction
