# Supplementary material for: Alpibectir–Ethionamide combination (AlpE) for the treatment of tuberculosis
Source: Nat Commun. 2026 Apr 7;17:4954. doi: 10.1038/s41467-026-71460-6 (PMC13234193; doi:10.1038/s41467-026-71460-6)
Supplement: Supplementary file 4 — Reporting Summary [file 41467_2026_71460_MOESM4_ESM.pdf]

nature portfolio

Corresponding author(s): Modesto J Remuiñán, Nicolas Willand, Michel Pieren, Alain R. Baulard

Last updated by author(s): Nov 14, 2025

Reporting Summary

Nature Portfolio wishes to improve the reproducibility of the work that we publish. This form provides structure for consistency and transparency in reporting. For further information on Nature Portfolio policies, see our Editorial Policies and the Editorial Policy Checklist.

Statistics

For all statistical analyses, confirm that the following items are present in the figure legend, table legend, main text, or Methods section.

n/a	Confirmed	
☐	☒	The exact sample size (n) for each experimental group/condition, given as a discrete number and unit of measurement
☐	☒	A statement on whether measurements were taken from distinct samples or whether the same sample was measured repeatedly
☐	☒	The statistical test(s) used AND whether they are one- or two-sided<br>*Only common tests should be described solely by name; describe more complex techniques in the Methods section.*
☒	☐	A description of all covariates tested
☐	☒	A description of any assumptions or corrections, such as tests of normality and adjustment for multiple comparisons
☐	☒	A full description of the statistical parameters including central tendency (e.g. means) or other basic estimates (e.g. regression coefficient) AND variation (e.g. standard deviation) or associated estimates of uncertainty (e.g. confidence intervals)
☐	☒	For null hypothesis testing, the test statistic (e.g. F, t, r) with confidence intervals, effect sizes, degrees of freedom and P value noted<br>*Give P values as exact values whenever suitable.*
☒	☐	For Bayesian analysis, information on the choice of priors and Markov chain Monte Carlo settings
☒	☐	For hierarchical and complex designs, identification of the appropriate level for tests and full reporting of outcomes
☒	☐	Estimates of effect sizes (e.g. Cohen's d, Pearson's r), indicating how they were calculated

Our web collection on statistics for biologists contains articles on many of the points above.

Software and code

Policy information about availability of computer code

Data collection	Concentration-response curves were read using the Ensight Multimode plate reader (PerkinElmer). Confocal microscopy images were acquired using the GE IN Cell 6500HS (GE Healthcare Life Sciences) automated confocal microscope. Next-generation sequencing was performed by an Illumina NextSeq 500 system. Samples for proteomics analysis were injected into an Ultimate3000 nanoRLSC (Dionex) coupled to a Q-Exactive mass spectrometer (Thermo Fisher Scientific). Thermal shift assays were conducted in an iCycler RT-PCR system (Bio-Rad, Hercules, CA) or in a CFX-Opus 96 Dx RT-PCR system (Bio-Rad CFX-Opus 96, Hercules, CA). Bacterial growth was measured using the BD BACTEC MGIT960 automated system for MIC testing when indicated.
Data analysis	Graph plots and statistical analyses were carried out using GraphPad Prism version 10.2.2 (GraphPad Software Inc.). Confocal microscopy images were processed using the Columbus image analysis software version 2.9.1 (PerkinElmer). Processing of transcriptomics data is detailed in the Methods section; tools used are Illumina quality control tools, PRINSEQ, Bowtie2, and SPARTA. For proteomics analysis, the instruments were operated with Tune 2.4 and Xcalibur 3.0 build 63. Mascot 2.4 (Matrix Science, Boston, MA) was used for protein identification. To solve the VirS-alpibectir crystal structure, the MOLREP program was employed using the VirS AlphaFold2 model as search template and the LigPlot+ program was used to map interactions between VirS and alpibectir.

For manuscripts utilizing custom algorithms or software that are central to the research but not yet described in published literature, software must be made available to editors and reviewers. We strongly encourage code deposition in a community repository (e.g. GitHub). See the Nature Portfolio guidelines for submitting code & software for further information.

Data

Policy information about availability of data

All manuscripts must include a data availability statement. This statement should provide the following information, where applicable:

- Accession codes, unique identifiers, or web links for publicly available datasets
- A description of any restrictions on data availability
- For clinical datasets or third party data, please ensure that the statement adheres to our policy

The electron density map and the coordinates of the refined crystal structure were deposited to the Protein Data Bank under PDB ID: pdb_00008RCX. Transcriptomics data were deposited in ArrayExpress and the accession code is E-MTAB-16253. The mass spectrometry proteomics data were deposited to the ProteomeXchange Consortium via the PRIDE partner repository with the dataset identifier PXD075165. All other data supporting the findings of this paper are provided within the main text, supplementary information, and source data files.

Research involving human participants, their data, or biological material

Policy information about studies with human participants or human data. See also policy information about sex, gender (identity/presentation), and sexual orientation and race, ethnicity and racism.

Reporting on sex and gender	NA
Reporting on race, ethnicity, or other socially relevant groupings	NA
Population characteristics	NA
Recruitment	NA
Ethics oversight	NA

Note that full information on the approval of the study protocol must also be provided in the manuscript.

Field-specific reporting

Please select the one below that is the best fit for your research. If you are not sure, read the appropriate sections before making your selection.

☒ Life sciences ☐ Behavioural & social sciences ☐ Ecological, evolutionary & environmental sciences

For a reference copy of the document with all sections, see nature.com/documents/nr-reporting-summary-flat.pdf

Life sciences study design

All studies must disclose on these points even when the disclosure is negative.

Sample size	No statistical methods were used to predetermine our sample sizes, which are considered as standard and are usually reported in publications.
Data exclusions	For the in vivo study, some mice were excluded from analysis due to culture contamination as indicated in Supplementary Table 7A. No data were excluded from other experiments.
Replication	Concentration-response curves were obtained using technical duplicates for each of 3 biological replicates. Transcriptomics and survival kinetics experiments were performed using 3 biological replicates. Proteomic studies were conducted in biological duplicate. For the in vivo drug efficacy studies, the untreated and pretreatment groups contained 12 mice each and the treatment groups contained 6 mice each. All attempts at replication were successful.
Randomization	Randomization was not considered for the in vitro studies. For the in vivo study, mice were randomly assigned to treatment and control groups.
Blinding	Blinding was not applicable to our study.

Reporting for specific materials, systems and methods

We require information from authors about some types of materials, experimental systems and methods used in many studies. Here, indicate whether each material, system or method listed is relevant to your study. If you are not sure if a list item applies to your research, read the appropriate section before selecting a response.

Materials & experimental systems

n/a	Involved in the study
x	Antibodies
	x Eukaryotic cell lines
x	Palaeontology and archaeology
	x Animals and other organisms
x	Clinical data
x	Dual use research of concern
x	Plants

Methods

n/a	Involved in the study
x	ChIP-seq
x	Flow cytometry
x	MRI-based neuroimaging

Eukaryotic cell lines

Policy information about cell lines and Sex and Gender in Research

Cell line source(s)	THP-1 cells were obtained from ATCC (ATCC TIB-202).
Authentication	None of the cell lines used were authenticated.
Mycoplasma contamination	Cell lines were not tested for mycoplasma contamination.
Commonly misidentified lines (See ICLAC register)	No commonly misidentified cell lines were used.

Animals and other research organisms

Policy information about studies involving animals; ARRIVE guidelines recommended for reporting animal research, and Sex and Gender in Research

Laboratory animals	4 week-old female Balb/c mice were used. All animals were grown in an animal facility under filtered air conditions (20-24°C), with a 12-hour light/dark cycle and relative humidity ranging from 45 to 85%, in plastic cages using sterilized wood shavings as bedding.
Wild animals	The study did not involve wild animals.
Reporting on sex	Only female mice were used; sex was not considered as a variable in the study design.
Field-collected samples	The study did not involve samples collected from the field.
Ethics oversight	The in vivo experimental project was favorably evaluated by the Charles Darwin Ethics Committee no. 005, located at Pitié-Salpêtrière Hospital (France), and clearance was given by the French Ministry of Education and Research under the APAFIS no. 12380-2017112809414820 v3. The animal facility was authorized to conduct animal experiments (license number C-75-13-08). The individuals involved in the animal experiments underwent specific training recognized by the French Ministry of Education and Research.

Note that full information on the approval of the study protocol must also be provided in the manuscript.

Plants

Seed stocks	NA
Novel plant genotypes	NA
Authentication	NA
